# Unconventional animal models of Alzheimer’s disease and aging: key insights from the UAMAA 2026 conference

**DOI:** 10.1038/s41684-026-01789-4

**Published:** 2026-09-16

**Authors:** B. Maximiliano Garduño, Tonatiuh Melgarejo, Patricia Cogram, Todd C. Holmes, Xiangmin Xu

**Affiliations:** 1 Department of Anatomy & Neurobiology, School of Medicine, University of California, Irvine, CA USA; 2 Department of Clinical Sciences, School of Veterinary Medicine, Western University of Health Sciences, Pomona, CA USA; 3 Department of Ecological Sciences, Faculty of Sciences, Institute of Ecology and Biodiversity, Universidad de Chile, Santiago, Chile; 4 The Center for Neural Circuit Mapping, University of California Irvine, Irvine, CA USA; 5 Department of Physiology and Biophysics, School of Medicine, University of California, Irvine, Irvine, CA USA; 6 Institute for Memory Impairments and Neurological Disorders, University of California Irvine, Irvine, CA USA

**Keywords:** Alzheimer's disease, Neural ageing, Cognitive ageing

## Abstract

Alzheimer’s disease (AD) remains a major unmet medical challenge, highlighting the need for improved experimental models. The second Unconventional Animal Models of Alzheimer’s Disease and Aging (UAMAA 2026) conference brought together ~150 interdisciplinary researchers to discuss advances across 19 animal models of AD and aging. Six major themes emerged: naturally occurring AD-like pathology, neuroinflammation, population heterogeneity, development of genomic and molecular resources, companion dogs as translational models and the promise of laboratory-amenable species such as the Chilean degu and common marmoset. These discussions highlighted the growing value of cross-species approaches for advancing translational AD research.

Alzheimer’s disease (AD) is the most common form of age-related dementia and accounts for an estimated 60–80% of worldwide dementia cases^1^. AD is an escalating health and financial crisis, a trend exacerbated by advances in medicine, technology and public health infrastructure that have lengthened the typical human lifespan. The epidemiological consequence of a rapidly aging global population is stark: over 55 million people currently live with dementia, a figure projected to triple by 2050 to over 139 million, with AD accounting for the overwhelming majority of that burden^2^. The economic toll reflects this trajectory: costs for AD and related dementias in the United States were estimated at approximately $450 billion in 2020 and are projected to surge to $3.3 trillion annually by 2060^3^. The human, economic and societal costs of this crisis cannot be overstated. AD is one of the most urgent unmet medical challenges of our time.

As a complex polygenic disease, AD has proven exceptionally challenging to treat and cure. Most AD clinical trials have failed, and even recent ones demonstrating disease deceleration carry serious side effects and show emerging evidence of sex-specific treatment outcomes^4^. One possible cause for this failure rate is the translational gap between basic science and the clinic. Several groups have posited an over-reliance on conventional animal models (e.g., rats and genetically engineered mice), a concern notably highlighted by Peter Libby in his commentary *Murine “Model” Monotheism*^5^. These incisive comments call for researchers to reflect on whether their questions would be better addressed by using a non-murine species that naturally exhibits age-related traits fundamental to the disease or biological mechanism they are studying.

The value of unconventional animal models (UAMs) has been recognized across numerous fields. Guinea pigs, like humans (but unlike rats and mice), cannot synthesize vitamin C, which made them critical players in the Nobel Prize-winning discovery of its biological importance. Ferrets, physically capable of vomiting unlike mice and rats, proved essential for developing anti-nausea drugs. Rabbits are prone to diet-induced atherosclerosis and were pivotal in the development of cholesterol-lowering statin drugs^6–8^. Despite these contributions, UAMs remain underutilized in neuroscience and AD research^9^.

Historically, several practical barriers have limited broader adoption of these models. Compared with mice and rats, many UAMs lack species-specific genetic tools, validated molecular reagents, reference genomes, cell atlases, standardized behavioral assays and well-established experimental protocols. Specialized housing, husbandry and breeding requirements further increase the cost and complexity of their use. In AD research, these challenges are compounded by the longer lifespans of many species, which are advantageous for modeling age-related neurodegeneration but require lengthy and expensive longitudinal studies. At the same time, decades of investment in murine models have generated extensive resources, infrastructure and community expertise, making conventional models the default choice for most laboratories. This imbalance has slowed the development of complementary resources for alternative species, despite their potential to provide unique biological and translational insights^10^.

In this context, the international conference on Unconventional Animal Models of Alzheimer’s Disease and Aging (UAMAA) was convened to highlight advances in emerging model systems, foster interdisciplinary collaborations and accelerate the development of the tools, resources and community needed to expand the repertoire of animal models for AD and aging research.

## UAMAA 2026: conference overview

The conference brought together researchers from around the world with expertise in UAMs, AD and aging. UAMAA 2026 was conceived as a special conference associated with the University of California, Irvine (UCI)’s Center for Neural Circuit Mapping Conferences^11^ and provided a forum to present emerging discoveries, foster interdisciplinary collaborations and accelerate the development of experimentally tractable and translationally relevant animal models for AD and aging research.

**Fig. 1:**
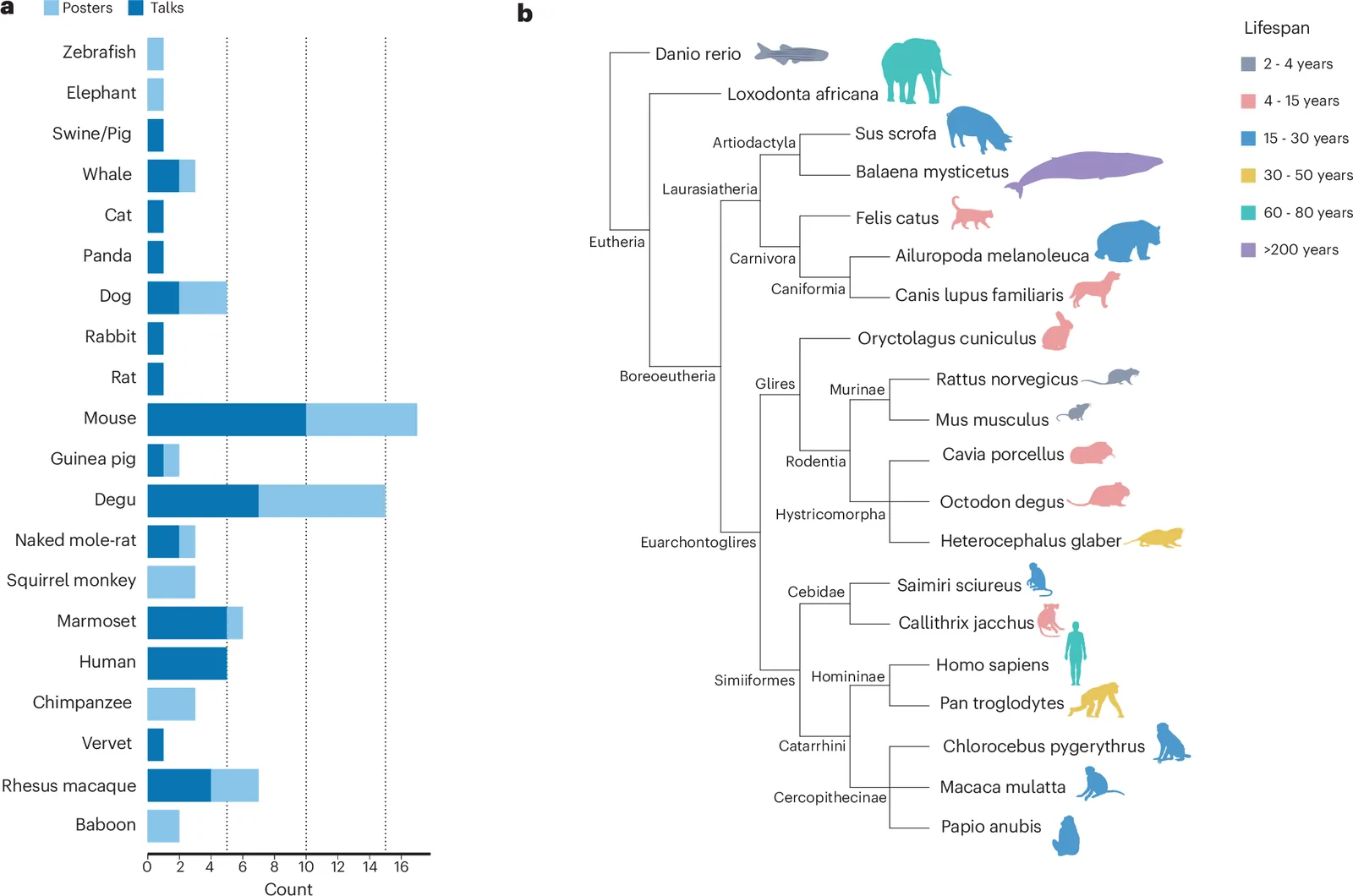
Species representation and phylogenetic breadth at UAMAA 2026. **a**, Horizontal stacked bar chart depicting the number of scientific presentations per animal model, stratified by presentation type: talks (dark blue) and poster presentations (light blue). **b**, Phylogenetic tree illustrating the evolutionary relationships among the 19 unconventional animal models and humans (*Homo sapiens*) represented at the meeting. Species illustration colors denote six groups based on typical lifespan. Species in (**a**) are vertically ordered to match the species in the phylogenetic tree (**b**).

Following the inaugural 2023 conference in Chile^12^, this second iteration aimed to build on that success by increasing the number of scientific leaders, animal models featured and the breadth of cutting-edge AD and aging research. The three-day event hosted over 150 attendees across 6 sessions featuring 37 talks, 30 poster presentations and 19 animal models of aging and AD (Fig. 1)^13^. A collaborative tone permeated the meeting, with new ideas, directions and collaborations naturally emerging throughout the talks, poster sessions and informal networking during breakfast, lunch and coffee breaks.

## Key Insights from UAMAA 2026

### Hallmarks of AD are naturally present across multiple mammalian species: a call to recalibrate translational research

**Fig. 2:**
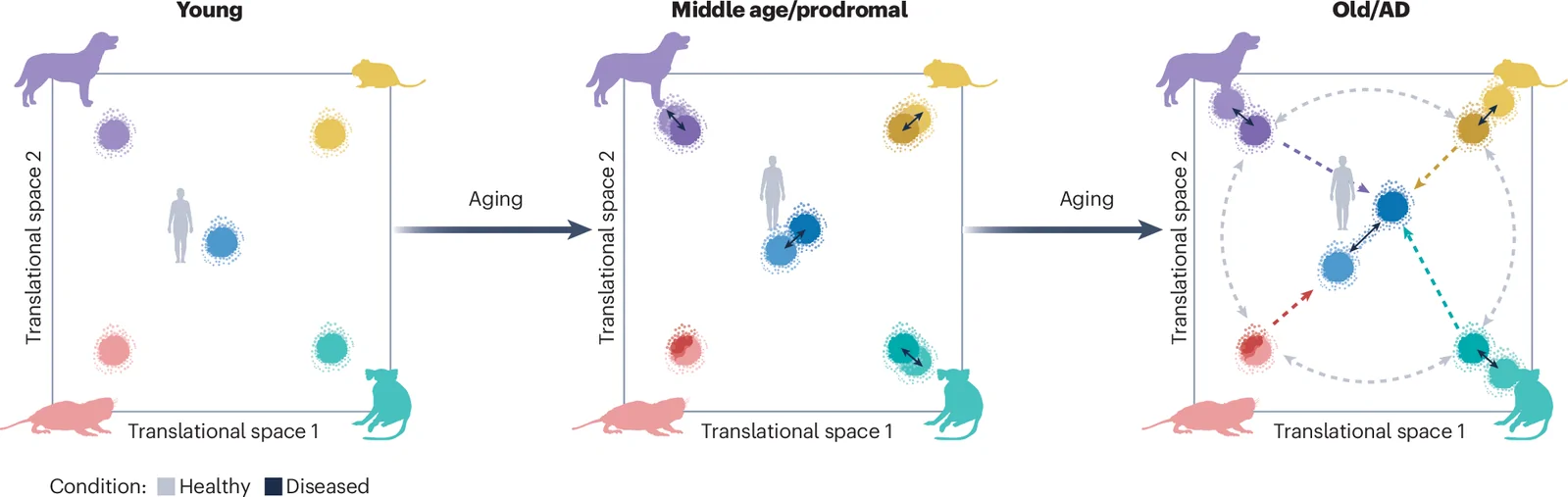
A theoretical translational space framework for unconventional animal models of AD and aging. Conceptual illustration depicting how four unconventional animal models—the Chilean degu (*Octodon degus*; yellow), common marmoset (*Callithrix jacchus*; green), domestic dog (*Canis lupus familiaris*; purple) and naked mole rat (*Heterocephalus glaber*; pink)—relate to humans (*Homo sapiens*; blue and gray) across aging (young (left), middle age/prodromal (middle) and old/AD (right)) within a theoretical ‘translational space’. Lighter shades represent healthy individuals, and darker shades represent AD-affected individuals. As animals age, populations begin to separate into healthy and disease trajectories (denoted by double sided black arrows), mirroring the heterogeneity observed in humans. Dashed arrows from the diseased degu, marmoset and dog clusters point toward the human AD cluster, illustrating each model’s attempt to model human sporadic AD. Dashed gray arrows between adjacent species highlight the potential for cross-species lateral optimization. A red dashed arrow from the naked mole rat cluster toward the healthy human cluster reflects its potential as a model of neurodegeneration resistance and healthy aging. All data are theoretical and intended to illustrate conceptual relationships only.

The conference showcased neuropathology across the mammalian clade, with degus, marmosets, dogs and vervets all developing AD-like features with aging. This convergence suggests that pathological features leading to neurodegeneration may be a conserved aspect of mammalian aging biology, rather than a uniquely human vulnerability. George E.P. Box’s aphorism “*All models are wrong, but some are useful”* holds particular weight here. What aspects of the AD-like traits observed in these species are translatable to human AD? Could these models inherently provide novel insights and therapeutic targets for the disease? Additionally, could these species be vehicles for lateral model optimization, whereby unique traits identified in one species inform and improve other animal models of AD, rather than serving solely as direct conduits for translation to humans (Fig. 2)? For example, if the degu rodent has a genetic profile capable of naturally manifesting AD-like traits, could some of their features be useful to refine existing murine models? If dogs reliably develop amyloid-β pathology while showing only limited and inconsistently reported neurofibrillary tangles, what can we learn and leverage from this model that naturally reproduces only part of the disease’s defining neuropathology? The answers to these questions could generate more incisive and translationally relevant targets, not only for treatment, but also for the development of next-generation animal models designed to better reflect the complexity of human AD (Fig. 2). Part of that complexity, as the conference made clear, lies beyond amyloid and tau, given that neuroimmune dysregulation has become an equally critical dimension of AD, raising unexpected questions about the very origins of the aging brain’s immune cells.

### Beyond microglial states: origins as an emerging factor in aging and AD

The neuroinflammation theme arose early in the conference and resonated across several subsequent talks, proposing that peripheral monocytes infiltrate the brain and replace resident microglial populations^14^. This evolving concept sparked discussion throughout the conference, with investigators raising the question of whether a similar dynamic could be occurring in their own models, including in cell culture models and xenotransplantation experiments in mice. Whether peripherally derived microglia are present across conventional and UAMs, how their presence relates to blood–brain barrier integrity and neuroinflammation, and whether they represent a viable therapeutic target emerged as compelling open questions. Furthermore, whether such infiltration occurs uniformly or varies among members of a species points to the importance of population heterogeneity in identifying vulnerable and resistant individuals.

### Embracing population heterogeneity as a scientific asset

Most UAMs do not have isogenic lines of animals readily available to investigators. Although population heterogeneity is often seen as a disadvantage in experimental design, it may also offer distinct advantages to aging and AD research. A heterogeneous animal population more closely mirrors the human population and encompasses a broader range of polygenic profiles to capture the genetic landscape of neurodegenerative risk. As in humans, a heterogeneous population can also produce resilient individuals, whose genetic makeup or environment provides protection against disease, a phenomenon observed across multiple UAM species. Studying the spectrum of aging phenotypes, from healthy to poor agers, within these heterogeneous populations, and how these compare to healthy and poor aging in humans, could provide valuable insights for the development of treatment and preventative strategies. Such diversity, however, demands tools, reagents and reference datasets that are robust enough to perform reliably across a genetically heterogeneous population, a foundational need that sparked much conversation throughout the conference.

### If the tool doesn’t exist, build it

One recurring motif of questions during lunch breaks was “how’s your model’s genome annotation?”, “does it have a reliable brain atlas?”, “do commercially available antibodies, reagents and protocols work for it?”. For a lot of these models, investigators are going into the unknown, having to build foundational tools that researchers working on conventional animal models typically don’t have to worry about. The UAMAA conference featured pioneers tackling these challenges head-on, building atlases, genome annotations, protocols and foundational datasets that will support the future of UAM research. Among these advances, a newly developed degu genome annotation, brain atlas and husbandry and behavioral protocols^15^ represented a substantial step forward for one of the most promising UAMs in aging and AD research. While these foundational resources are actively being built for many UAMs, one model, the companion dog, stands apart entirely, as it is not maintained in a laboratory setting and offers a unique parallel to the human aging process.

### Companion dogs represent a compelling translational platform for AD research

Dogs naturally develop canine cognitive dysfunction (CCD), a spontaneously occurring syndrome with clinical and neuropathological features that closely resemble human AD. What uniquely distinguishes companion dogs from other animal models is their shared living environment with owners. Unlike laboratory animals housed in controlled facilities, companion dogs inhabit human households and are exposed to the same complex environmental and lifestyle factors, which collectively shape aging trajectories and disease susceptibility. Recent untargeted plasma metabolomic profiling of companion dogs with CCD has revealed systemic biochemical alterations mirroring those observed in human AD, providing molecular evidence of this model’s translational validity^16^. Together, these advantages position companion dogs as a unique bridge between the environmentally embedded complexity of human AD and highly controlled laboratory models, including promising UAMs of sporadic AD.

### Degus and marmosets emerge as frequently discussed UAMs for sporadic AD research

Among the diverse models featured in the conference, there were two notable standouts: the Chilean degu (*Octodon degus*)^17^ and the South American marmoset (*Callithrix jacchus*)^18^. Both animals have relatively long lifespans (5–9 years and 10–14 years, respectively) and naturally develop several age-associated AD-related pathological features. They are medium-sized (200–260 g and 230–260 g, respectively) with relatively low husbandry costs, exhibit complex behaviors and have proven amenable to breeding, handling and experimentation in the laboratory.

While these species share compelling common ground, key distinctions deserve mention. Degus offer meaningful practical advantages over marmosets: lower acquisition and husbandry costs, fewer regulatory and cultural barriers (as rodents are subject to less stringent oversight than nonhuman primates), faster reproductive cycles (90 days compared to 143 days in marmosets), earlier recommended breeding age (6 months compared to 18 months in marmosets) and notably larger litters (5–7 compared to 2–3 in marmosets). Collectively, these features lower the barrier to entry into the research space^15,19^. As rodents, degus are more closely comparable to mouse and rat models, enabling direct cross-species comparison and allowing researchers to leverage the vast body of murine AD research as a reference framework. Marmosets, on the other hand, bring their own notable scientific merits: transgenic AD-relevant lines are available; marmosets are phylogenetically closer to humans and their brain architecture better resembles that of the human brain. Together, these complementary flagship species from the rodent and primate orders represent exciting experimental platforms for investigating sporadic AD. Far from competing with one another, they are collectively sparking fresh ideas, energizing the field and expanding the experimental toolkit available to AD researchers.

A third species deserving mention is the naked mole rat, which captivated conference attendees not for its AD pathology, but for its extraordinary resistance to neurodegeneration and capacity for healthy aging, raising compelling questions about what protective mechanisms from the naked mole rat could be harnessed therapeutically. More broadly, the 19 models/species represented at UAMAA 2026 each offered distinct perspectives on AD pathology, resilience and aging, underscoring the breadth of the field and a simple yet profound truth: from the dog sharing our home to the massive Bowhead whale navigating the open ocean, every one of these animals ages and each holds lessons about vulnerability, resilience and the biology of growing old.

## From unconventional to essential: a new translational vision

The UAMAA 2026 conference showed that UAMs provide valuable insights into aging, AD and resilience and help to advance the field by notably improving the modeling of complex polygenic diseases inherent to human aging. The widespread implementation and optimization of genetic engineering in mice have produced vast amounts of invaluable data that have improved our understanding of the biology and treatment of human disease. However, current transgenic murine efforts to model human AD have limitations. Several reasons have been speculated to contribute to this translational gap, from the lifespan of mice being too short (~2 years) to develop AD-like features, to their quick generational turnover times, which may have contributed to accelerated phylogenetic divergence from humans^20^. What is clear, however, is that there is an urgent need for models that better recapitulate human sporadic AD. The field has rapidly adopted multi-modal, cross-disciplinary and multi-institutional research programs to address AD. All available evidence suggests that we are now at an inflection point, where multi-organismal approaches should be included (Fig. 2). The meeting showed how many of the barriers to entry for these “unconventional” models, such as the lack of genome annotations, reagents and brain atlases, are readily surmountable, opening novel avenues for research and discovery. The UAMAA conference will continue to serve as a platform and community anchor to push forward this new vision aiming to increase the translational relevance of AD and aging research, with the overall mission of promoting healthy aging and preventing human brain disease. The third conference, to be hosted by the University of Pittsburgh in 2028, is a testament to the growing momentum and expanding reach of this international community.
